# Counting the costs: understanding the extra costs of living with disability in Indonesia to advance inclusive policies within the SDG framework

**DOI:** 10.3389/fresc.2024.1236365

**Published:** 2024-10-22

**Authors:** Irma Marlina, Ginanjar Wibowo, Desi Dwi Bastias, Bimbika Sijapati Basnett, Dinar Dwi Prasetyo, Mercoledi Nasiir

**Affiliations:** ^1^ Center for State Budget Policy, Fiscal Policy Agency (BKF), Ministry of Finance, Jakarta, Indonesia; ^2^ Gender Equality, Disability, and Social Inclusion Team, Australia Indonesia Partnership for Economic Development (Prospera), Jakarta, Indonesia

**Keywords:** sustainable development goals, disability inclusion, extra costs of living with disability, indonesia, disability concessions

## Abstract

The Sustainable Development Goals (SDGs) are a multidimensional framework for monitoring progress on disability inclusion over time and among countries where reliable, disability disaggregated data is available. However, the SDGs alone do not provide insights into the causes of the social and economic disparities that people with disabilities face or offer specific policy solutions to alleviate them. This paper highlights the extra costs of living with disability in Indonesia to advance the country's commitment to further the rights of people with disabilities. It utilizes three primary estimation methods, combining an analyses of national survey data with primary data from interviews and focus group discussions. Findings reveal significant and varying costs based on disability type, severity and life cycle stages. It also highlights the unaffordability of these costs for most individuals with disabilities and their families. Leveraging these estimates, the paper proposes ‘disability concessions’ aligned with Indonesia's legal framework on disability inclusion, aiming to alleviate financial burdens through discounts across health, education, utilities and transportation. By contributing to methodological approaches in understanding extra costs of living with disability inclusion in emerging country context and promoting discussions on leveraging the results for disability inclusive policymaking, this paper supplements the SDG framework to foster disability inclusion.

## 1 Introduction

The recognition and promotion of disability rights are integrated as disability inclusion in the Sustainable Development Goals (SDGs). The SDG framework encompasses seven targets explicitly focusing on persons with disabilities, complemented by an additional six targets and universal targets with the SDGs can also be disaggregated by disability (1). By encompassing these targets and indicators, the SDGs illuminate the profound inequalities experienced by individuals with disabilities across various aspects of life, including in education, labour market, poverty outcomes, public services and more. These inequalities are also evident in Indonesia (2, 3). Additional analysis is also required to understand the underlying causes of these disparities and develop effective policy solutions.

Understanding the higher costs of living experienced by individuals with disability is a requisite step for developing effective policies and promoting inclusivity (4–7). Having a disability entails direct and indirect costs for individuals and their families, including expenses for essential items and disability-specific needs, as well as foregone income and reduced employment opportunities. From a policy perspective, quantifying these costs is needed for resource allocation decisions and enabling inclusivity. Estimating these costs also enables evidence-based policy decisions and the assessment of intervention effectiveness.

Although research on estimating the extra costs of living with disabilities exists, it is predominantly concentrated in high-income countries. However, contextual factors (such as availability of services, accessibility of the broader environment, level of awareness) emphasize the need for studies in emerging and low-income countries. Encouragingly, there is a growing trend of extra cost research in emerging countries, with notable examples including Vietnam (8), several countries in Africa (9), China (10), Philippines (11), and Georgia (12).

This study, conducted collaboratively by researchers from Indonesia's Fiscal Policy Agency of the Ministry of Finance and the Australia Indonesia Partnership for Economic Development (Prospera), contributes to the expanding body of research by examining the additional costs associated with living with disabilities to inform policy solutions for inclusivity in Indonesia. Using three primary estimation methods and combining an analyses of national survey data and primary data collected with persons with disabilities, it highlights extra costs and variations of costs based on disability type, severity, household poverty status and life cycle processes.

The study's findings were instrumental to shape our proposal for disability concessions package as mandated by Indonesia's disability Law No. 8/2016. The estimated extra costs were used to justify the level of each proposed discounts. Informed by findings from consultations and benchmarking with other countries, the study recommended discounts on essential goods and services in four priority sectors, including education, health, transportation, and utilities. The benefits include top-ups on education allowance, subsidies for health insurance premium, assistive devices and rehabilitation services, transportation ticket discount, and tap water, electricity, and internet discounts. These packages are designed to alleviate the financial burden on individuals with disabilities and their families.

The paper is structured into four sections: background and literature review, methodology, research findings on extra costs analyses and its role in designing disability concession and concluding lessons and future research and policy agenda.

## 2 Socio-economic profiles of persons with disabilities in Indonesia & the study on “extra costs”

Indonesia, the world's largest archipelago with 17,000 islands and a land are of 1.9 million square kilometres, is home to 274 million people, making it the fourth most populous country globally. Administratively, Indonesia is divided into 34 provinces, with numerous regencies and cities encompassing over 6,000 inhabited islands. The population is divided between urban and rural areas, with a 57.4% residing in urban regions, particularly in Java where major cities are located. Urbanization has been a prominent trend, with a growing percentage of population residing in urban centres. However, there is still a substantial population residing in rural communities. The diverse administrative and population distributions present complex context for studying the extra cost and developing inclusive policies to address the challenges faced by individuals living with disabilities (13).

Government surveys and censuses in Indonesia play a crucial role in gathering social and economic data, providing insights into the disability related goals in the SDGs. Since 2015, Statistics Indonesia (BPS), Indonesia's non-ministerial government institution in charge of providing national data, has incorporated the disability identification instrument from the Washington Group Short Set questionnaire into the Inter-Censual Population Survey (Supas), which is conducted every ten years. Supas is released in alternate with the National Census. Supas serves as a main reference to estimate the headcount, profile, and distribution of persons with disabilities due to its large sample size allowing for subnational disaggregation. Furthermore, annual surveys with smaller samples like the National Labor Force Survey (Sakernas) since 2016 and the National Socioeconomic Survey (Susenas) from 2017 include questions adapted from the Washington Group Short Set, enabling analyses of labour and socio-economic outcomes. Nevertheless, it is important to note that there are challenges regarding data comparability. The annual surveys in Indonesia are not specifically designed to capture disability, and certain disability related questions are not consistently included. Thus, we refer to data from different time periods in our analyses.

Around 2.1% of Indonesia's population, or approximately 6 million people, have moderate to severe disability, according to our analyses of the Supas 2015 data. Women in Indonesia have a slightly higher prevalence of disability (2.3%) compared to men (1.8%). 63.4% of Indonesians with disability are below 65 years old, highlighting the importance of addressing disability inclusion issues among children and working-age population. People with disabilities are spread across the country, as highlighted in Supplementary Figure S1. Nevertheless, several provinces in Java and Sulawesi stand out with slightly larger disability prevalence, with Yogyakarta province (2.9%) and Central Sulawesi (2.5%) exhibiting a relatively higher prevalence of disability when compared to others.

Approximately 50% of individuals report having a single disability, while the other 50% reporting having multiple disabilities (refer to Table 1). Mobility (40%) and vision (37.3%) are the most reported types of disabilities, with no observable gender differences in reported types of disabilities. There are notable gender differences in prevalence of single or multiple disabilities. Almost 70% of men with a disability report single disabilities whereas 53% of women report multiple, suggesting that women with disabilities are more likely to experience multiple disabilities when compared to men. This underscores the need to apply a gender lens to address unique challenges faced by individuals with multiple disabilities, such as coordination of care and specialized services.

**Table 1:** Types of disability, 2015.

Number of difficulties	Type of functioning	Percentage	Total
Single	Seeing	18.4%	56.8%
	Hearing	9.1%	
	Walking	18.1%	
	Remembering/Concentrating	7.0%	
	Communicating	2.4%	
	Self-care	1.9%	
Multiple	With Seeing	32.0%	43.2%
	With Hearing	31.0%	
	With Walking	47.8%	
	With Remembering/Concentrating	33.9%	
	With Communicating	23.1%	
	With Self-care	22.4%	
Total			100.0%

The percentages indicate the prevalence of specific types of functioning difficulties among individuals with moderate to severe disabilities. In cases of “multiple”, or more than one type of functional difficulties, the percentages may not sum to 100% due to potential overlapping of functioning categories. The denominator for “multiple difficulties by type of functioning” is the total count of individuals with multiple difficulties. Shading is provided as a visual cue to the proportion order.

Author's calculation from Supas 2015—Indonesian government benchmark.

Persons with disabilities in Indonesia face significant disparities in education, labour market and poverty outcomes. The data presented in Supplementary Figure S2 highlights a significant and widening gap in educational completion rates between young adults with disabilities and their counterparts without disabilities, particularly when considering the severity of disability. In 2022, the 12-year school completion rate for young adults without disabilities was 56.5%, compared to 50.6% for individuals with disabilities, with the lowest rate observed among those with severe disabilities. This represents a 17% gap in completion rates between individuals with severe disabilities and the general population, likely influenced by various barriers (such as inadequate support systems, inaccessible learning environments, limited availability of specialized resources) and cumulative disadvantages (such as communication difficulties, learning disabilities). Such limited educational completion rates are concerning as they can have long-term implications for employment prospects, economic independence, and the perpetuation of socio-economic inequalities by disability status, as is reflected in their labour market participation gaps below.

In 2022, the labour force participation rate for individuals with no disability was 70.6%, while for those with disabilities was only 44.9%, with the lowest rate observed among people with moderate/severe disability at 19.5% (Supplementary Figure S3). When employed, people with disabilities often earn less, with an average monthly income 40.5% less than the national average of IDR 2,255,465 (≈USD 152.06 in August 2022). These disparities can be attributed to a higher proportion of individuals with disabilities working in informal sector or being self-employed, as well as limited job opportunities. The lower employment rates and income disparities highlight the challenges individuals with disabilities face in the labour market, which is exacerbated by educational gaps. A recent report by the International Labour Organization reveals wage/earning gap between individuals with disabilities and those without decreases as level of education increases, highlighting the importance of addressing educational disparities in improving labour market outcomes for individuals with disabilities (14).

Furthermore, the presence of disability within a household can have implications for other household members who do not personally experience disability. Data from 2018 shows more than 40% of people living in households with a member with disability are out of the labour force, double the figure of households with no disability (Table 2). This suggests that the impact of disability extends to the labour force participation of other household members, likely due to their added care responsibilities they have to assume.

**Table 2:** Labour force (LF) status of other household members, 2018.

Type of activity	No disability	Lower threshold disability (slight/moderate/severe)	Higher threshold disability (moderate/severe)
Within LF—working	67.8%	58.3%	49.3%
Within LF—not working	2.9%	2.9%	2.7%
Outside LF—student	8.4%	6.0%	4.6%
Outside LF—homemakers	18.9%	22.5%	22.8%
Outside LF—others	2.0%	10.4%	20.6%

Author's calculation using Susenas data, March round, 2018.

Households with a member with disability in Indonesia generally experience higher levels of poverty compared to their counterparts with no disability. Between 2018 and 2022, the disability-disaggregated estimated poverty rates consistently showed a 1–2 percentage point (pp) higher for people with slight to severe disability, and 5–6pp higher for those with moderate to severe disability (Supplementary Figure S5). These should be interpreted with caution, as poverty estimates require adjustment for disability-related extra costs, as we will discuss in the next section.

In 2022, households that have a member with a disability are almost five times more likely to report transfers (either cash or in-kind) as a means to fund their expenditure, compared with households with no disability (15.5% compared with 2.9%). These figures are concerning as they highlight the financial precarity faced by families that have a member with disabilities likely face.

Certain SDG targets and indicators, using current socioeconomic survey data, can provide insights into disparities faced by people with disabilities in education, poverty, and labour market. For instance, SDG target 8.5 focuses on equal opportunities and non-discrimination in employment for all, with Indicator 8.5.1 tracks employment-to-population ratio of persons with disabilities. SDG target 4.5 emphasizes equal access to quality education for all individuals, including people with disabilities. Target 1.1, which calls for eradication of extreme poverty by ensuring that all people have access to basic services, social protection, and economic opportunities, can also be disaggregated by disability. While these targets and indicators have provided a basis for monitoring the state of people with disabilities, they offer limited insights into underlying reasons behind these disparities. Complementary frameworks are thus needed.

Recognizing the extra costs that individuals with disabilities bear to maintain a similar standard of living as those without disabilities, and gaining a better understanding of these costs, is essential for effectively and systematically addressing the challenges that people with disabilities face. As mentioned in Section 1, people with disabilities incur additional direct and indirect costs that can be substantial and vary based on severity and type of disability, as well as other socio-economic characteristics of individuals and their families. Recognizing and quantifying these costs is needed to enable informed policy making and resource allocation to support individuals with disabilities and reduce financial disadvantages (7).

## 3 Methodological approaches to estimating extra costs of living with disabilities and their application to Indonesia

A systematic review of peer reviewed journal articles on extra costs of living with disability conducted by Mitra et al. (6) identifies three main approaches used in measuring these costs. The first approach is the “standard of living” approach, which compares the spending patterns of households with and without a member with disability using national socio-economic survey data. The second approach, known as the“goods and services” approach, focuses on the additional expenses incurred by individuals with disabilities, based on information on specific goods and services they purchase for basic participation in society. And “goods and services required” approach measures the extra costs required by persons with disabilities by collecting information on what expenditures would be needed to enable them to participate in society. Neither approach holds a clear advantage over the others, as each method offers unique insights into disability extra cost. This section provides an overview of each method and explains how they have been adopted for the purposes of this study in Indonesia, summarised in Table 3.

**Table 3:** Comparison summary of the three methods to measure Indonesia extra cost.

Compared traits	Method		
	Standard of living	Goods and services (GS)	Goods and services required (GSR)
Means of gathering data	Econometric exercise using secondary national dataset (indirect)	Survey to diverse profile of people with disability (direct)	FGD to a panel of professionals on disability issues and people with disability (direct)
Types of disability covered	6 types of functioning (Washington Group/WG Short Set questions)	8 types of functioning (pre-determined types) that can be classified to 6 types of functioning (WG short set questions)	8 types of functioning (pre-determined types)
Number of samples	315,672 households representing 71.4 million households nationally	80 people with disability representing 8 types of disability living in various areas and 3 age group + 8 typical people living in various areas and 3 age group	23–30 people with disability and caregivers, representing 8 types of disability
Results obtained	Average costs of disability incurred, in terms of proportion relative to typical Indonesian	Average unit costs of disability, in monetary terms, disaggregated by demographic and type of services	Cost items of goods and services relevant for disability

Author's documentation from GS and GSR study.

The “standard of living” (SOL) approach posits that households with similar income and characteristics but differing only in the presence of a member with a disability, should have comparable standards of living. If there is a disparity in their wealth, it is attributed to extra costs associated with disability, which hinder the accumulation of assets and contribute to the wealth gap between these households. The approach is used to measure the difference in observed material well-being by disability status, using data obtained from national socioeconomic surveys. This approach is also referred to as the “expenditure equivalence” approach.

Its underlying logic relies on the shared ordinality of common household's both income and standard of living, assuming constant needs. If a household has greater needs than the average household, their standard of living may be lower. In the case of households with a member with disability, the gap in the standard of living is considered the cost of living with disability.

This approach is elaborated in Zaidi & Burchardt (15), along with the breakdown of the theoretical groundwork. Algebraically the estimate can be written as:

S=αY+βD+γX+k

Here, *S* represents an indicator of standard of living, *Y* is household income, *D* is disability status (whether there is a member with disability in the household), *X* is a vector of other relevant characteristics, and *k* is the intercept term expressing a constant absolute minimum level of standard of living.

The extra cost of disability, *E*, is derived from the above equation and can be calculated as:

$$E=\frac{dY}{dD}=-\frac{\beta }{\alpha }$$

This value represents the “cross elasticity” of having a member with a disability. In other words, it is a portion of income/expenditure that is not considered when assessing overall wealth, as it is reallocated for meeting the disability-related costs.

The SOL approach is commonly used due to its cost-effectiveness, utilizing existing national datasets with disability identifier. It can identify where basic participation, such as school enrolment and work participation, is needed. Furthermore, by measuring the current economic impact of disability related expenditures, it provides a more accurate depiction of living conditions and poverty exposure.

However, it is important to note that the results obtained using this approach can vary significantly across studies (6, 9, 16). One main reason is because of the absence of a strict protocol for defining the variables used in the econometric exercise, including the construction of the standard of living measure itself (4). There have been instances where counterintuitive findings, such as non-significant or negative results, have been reported (9).

The “goods and services” (GS) approach, also referred to as “goods and services used” approach (17) and “expenditure-based” approach (18), directly measures the monetary expenses incurred by individuals with disabilities for their participation in society and economy. It typically encompasses expenses related to self-care, going to school/work, accessing healthcare facilities and shopping for essential goods. It is usually administered through questionnaire or diaries to people with disability.

While the approach is effective in identifying expenses relevant to disabilities, it relies heavily on the knowledge of surveyed individuals, which may result in lower reported expenses if either awareness of their needs is limited, or they do not have resource to purchase the goods. In some cases where “goods and services” is compared directly to their non-disabled counterpart, it may introduce bias by underestimating the actual cost of disability (4). People with disabilities may use the items being measured less frequently than their non-disabled counterparts due to the additional costs they incur to participate at the same level. In extreme cases, individuals with disabilities may rarely or never participate in the activities being measured, while those without disabilities participate much more. Consequently, the estimated expenditure associated with living with a disability may appear to be very low or even negative.

Lastly, the “goods and services required” (GSR) approach gathers information on what expenditures would be required to enable a person with disability to engage equally in basic social and economic activities, which may include those that they are currently not involved in. This method takes into account actual expenses and unmet expenses. For instance, in relation to children with disabilities attending school, it helps measure the additional associated costs, such as transportation and assistive devices. It also provides a measure of the goods and services that would be required for a child of school age, even if they are not currently attending school. It is expected that the extra costs required for goods and services would be higher compared to those for used goods and services. Wilkinson-Meyers et al. (18) provide a detailed explanation of this approach, although they do not explicitly refer to it as “goods and services required”. They use a mixed-method approach, combining the identification of relevant activities through focus groups with experts and validating the findings through a survey.

According to Mont, Cote, et al. (7), this method involves assembling a team of experts, including service providers, researchers, people with disabilities and organization representing persons with disabilities. They design an economic questionnaire with a comprehensive list of goods and services needed by disability subgroups (types of disabilities, gender, age group, work status). Focus group discussions are conducted with people with disabilities to gather more information, validate and expand the initial lists. The expert panel modifies the lists based on focus group data, assigning costs ranges considering disability severity and infrastructure availability. Market research may be conducted for unavailable or unpriced items. Updated prices are incorporated into spreadsheets, providing cost information by disability group, support needs, participation levels and time frames. The method's limitation is its lack of precision due to diverse needs and estimation challenges. However, it effectively identifies cost areas, guides service design, highlights insufficiency of simple cash benefits, and stimulates discussion on addressing extra costs.

Using the “standard of living”, “goods and services” and “goods and services required” approaches as complementary methods in estimating direct costs of disability is a preferable approach, considering the strengths and limitations of each approach (Table 3). In the case of Indonesia, all three approaches were employed to gain a better understanding of the direct extra costs associated with living with a disability. The study was conducted from June 2020 to February 2021, with the steps outlined below and the utilisation process of all study elaborated in Supplementary Figure S4.

### 3.1 “Standard of living” (SOL) approach

Our calculation is based on Susenas data, March round, 2019, Indonesian national dataset collected annually to collect information on Indonesian's basic characteristics and their socioeconomic outcomes. This dataset also collects information on disability status, using Washington Group short set questions. With samples reaching up to 320,000 households (approximately 0.45% out of the estimated national number), this dataset has served to be the main reference for poverty figures.

Susenas dataset does not collect income, instead it collects information on expenditure. In Indonesia, more than half out of all jobs are classified under informal sector that does not guarantee a steady income.^1^ Such limitation makes information on income harder to collect relative to expenditure. Expenditure may also be used to measure standard of living, although its purpose is slightly different with income in nature. Consumption measures the currently achieved standard of living of a household, whilst income measures the potential standard of living (20).

The econometric exercise in our study followed Zaidi & Burchardt (15) model with a slight adjustment. For the dependent variable, we measured the standard of living by constructing an asset index made out of 16 asset items ownership and private dwelling structures. The index is modified to have a range between 0 (indicating no asset ownership) to 100 (owning all assets). The independent variables are as follows: (1) Measure of income was substituted with per capita expenditure adjusted with Indonesian poverty line, in order to rule out the variation of cost of living in different regions; (2) Measure for disability was defined as a binary variable of having a member with a type of disability, based on Washington Group Short Set questions; (3) Vector of controls was made by including variables relevant for explaining household demographics. The regression was done on household level, and after several attempts we found that regressing separately by type of disability and expenditure quintile produced the most robust result. More detailed technical explanation on the exercise and the complete result of the econometric exercise is available in the Appendix section.

The main limitation of this method comes with regards to estimating the costs for multiple disability. Although they represent half of the population with disability, our regression approach that singles out each type of disability does not allow for calculating multiple-type disabilities.

### 3.2 “Goods and services” (GS) and “goods and services required” (GSR) approaches

The execution of GS and GSR were concurrently employed throughout October 2020–February 2021 (refer to Supplementary Figure S4 for the sequential process). The process began with consultations with international experts from Center for Inclusive Policy and national research partners at SIGAB (an organization representing people with disability in Indonesia) in late September 2020 to establish the study design and develop a list of required supports by different age groups (young, productive and elderly) and 7 disability types (vision, hearing, mobility, psychosocial, concentration and remembering, communicating, self-care/advanced physical disability) along with multiple disabilities (people with deafblindness, severe cerebral palsy, spinal cord injury, among others).

The first sequence is through GSR initial data collection, involving 4 focus group discussions with 30 disabilities experts and caregivers who are members of organisations of people with disability. This sequence focused on determining the basic needs of people with disabilities across the following domains of participation: daily self-care at home, access to health, access to shopping, going to school, going to work, and social life. Since the study was undertaken during the peak of the COVID-19 pandemic, we also distinguished between what was the case before and during the pandemic, to be able to isolate impacts of such an unprecedented event. Inputs from the FGDs were used to refine the survey instrument, which was benchmarked against similar studies undertaken in Bangladesh (21) and South Africa (22).

The second sequence is the GS survey, administered to people with disabilities and a smaller sample of those without disabilities in Yogyakarta Province, between October and November 2020. Yogyakarta was selected due to its high disability prevalence, availability of disability related services and under the SIGAB operating domain, which made it easier to identify respondents and administer the survey during the pandemic situation. The survey involved 80 individuals identified through snowballing method with various ages, types, and severities of disabilities, as a well as a comparison group of individuals without disabilities who shared similar demographic characteristics. The survey instrument included household socio-economic situation (income, support received, food security and utilities); information about individuals within the household (e.g., education, paid and unpaid activities); disability identification questions following the Washington Group Short Set; and the costs to access six different activities listed. We provided an extensive list of assistive technology, support (e.g., human assistance, sign language), mode of transportations that are used/required by people with disabilities. The collected data was then digitalized, cleaned, and analysed.

Third sequence is the GSR validation session, involving 23 representatives of organisations with disability. These people are either disability expert or are persons with disability themselves, and most of them were involved in the GSR initial focus group discussion data collection. The main objective of this session was to validate the cost estimates obtained from the survey, not only within the context of Yogyakarta but also in other regions where the experts are located. This helped ensure the accuracy and applicability of the findings across different regions. In addition, we also sought expert's perspectives on policy actions that could be taken to reduce the main cost drivers we identified. By engaging the disability experts in this way, we were able to enhance the credibility and relevance of our findings and ensure the proposed interventions, which we will outline in Section 4, were informed by expertise and experiences representing or working closely with individuals living with disabilities.

We encountered several issues during the study design and implementation. First, due to the lack of database on people with disabilities, we were unable to survey more individuals or select respondents randomly. Instead, we relied on snowball sampling from experts and representatives we consulted in the initial FGDs. This allowed for diverse representation based on gender, age and eight types of disabilities. However, due to the limited sample size, we could not differentiate respondents by the level of support or areas of residence (urban/rural) as recommended by the GSR guidelines by Wilkinson–Meyers et al. (18).

## 4 Results and analyses: heterogeneity of extra costs & their drivers

Our study findings across the three estimation methods indicate that individuals with disabilities in Indonesia experience significantly higher living cost compared to those without disabilities. These costs vary depending on the type and severity of disability, as well as demographic factors. However, the key finding is that monthly costs identified in this study are unaffordable for most individuals and families in Indonesia. The main factors driving these costs include the requirement for human assistance, transportation, assistive devices and additional health care.

Results from the “standard of living” approach, regressed separately by quintile and type of disability, show that the estimated per capita extra cost goes up to 20 percent more than typical per capita expenses, per each type of disability (Table 4). Extra cost is more likely to be significant and prevalent for households with moderate to severe disability, as opposed to those of slight to severe. Type of disability also matters for extra cost, with certain types of disabilities incurring higher extra costs compared to others. Amongst the moderate to severe disabilities, those with communicating bear the lowest cost (16.4–16.9%) while individuals with sight and hearing disabilities could face the highest cost (up to 22.4%).

**Table 4:** Summary of extra cost result from SOL regressions by specification, 2019.

Specifications		Extra cost	
Type	Quintile	Slight/ moderate/ severe	Moderate/ severe
Sight	Q1 (Poorest)	2.9%	4.9%
	Q2	4.3%	9.1%
	Q3	6.0%	12.0%
	Q4	7.4%	16.2%
	Q5 (Richest)	12.4%	22.4%
Hearing	Q1 (Poorest)	4.7%	8.3%
	Q2	5.7%	9.4%
	Q3	9.0%	11.0%
	Q4	9.4%	9.3%
	Q5 (Richest)	14.3%	21.9%
Mobility	Q1 (Poorest)		
	Q2		6.1%
	Q3		
	Q4	8.5%	8.8%
	Q5 (Richest)	11.8%	19.6%
Remembering/ concentrating	Q1 (Poorest)		
	Q2	13.2%	9.7%
	Q3	9.3%	17.6%
	Q4	13.4%	13.5%
	Q5 (Richest)	22.1%	18.6%
Communicating	Q1 (Poorest)		
	Q2	11.4%	19.4%
	Q3		11.7%
	Q4	7.6%	16.4%
	Q5 (Richest)	19.8%	16.9%
Self-care	Q1 (Poorest)		
	Q2	11.4%	
	Q3		
	Q4	7.6%	
	Q5 (Richest)	19.8%	18.4%

Blank cells indicate disability status of the household (and therefore extra cost) as statistically not significant (*α* > 10%). Results in bold indicate the highest cost amongst the corresponding type of disability. The complete result of the econometric exercise is available in Appendix.

Author's calculations using Susenas 2019.

The result found that higher income groups tend to face higher extra costs for disabilities across different types of disability (Table 4). Moreover, the lower income groups are more likely to face lower or even non-significant extra cost, suggesting their limitation in accessing services or other supports needed for their impairment. Lastly, even though the extra cost rises as the quintile goes up, the progression is not strictly linear. The findings are in line with empirical insights found in the other countries and contributes to the ongoing debate of the non-linear trend of extra cost across standard of living level (9, 15, 23).

In the Method section, we discussed how the result comes in terms of proportion of expenses, or “multiplier” to the expenses incurred by typical person. Such result is not easy to grasp on first view. To highlight the key message, we applied the relevant multipliers to Indonesia's official poverty line. The poverty line indicates the minimum amount of money needed to cover basic monthly needs.^2^ We chose multipliers that are calculated from households with a member with single-type disability living in the bottom 40% of the economy, which are the poor and vulnerable population. For households with a member with multiple disability, we chose the highest multiplier out of all the disability types. Such strategy helped us better understand how disability impacts a household's welfare and poverty. By applying the strategy, we find that almost 1 in 4 households with moderate to severe disability could be considered poor under the adjusted poverty line. In comparison, only 1 in 12 typical households (with no disabilities) were considered poor (shown in Supplementary Figure S6). This highlights how having disability significantly increases the risk of co-experiencing poverty.

### 4.1 SOL result

The results obtained from the SOL approach highlight the significant extra costs faced by Indonesians with disabilities, as well as the variation in costs depending on the type of disability and quintile group (wealth status of households). However, these results are general in nature and do not capture the specific cost drivers behind the estimated multiplier. Furthermore, the multiplier itself does not provide an accurate representation of the actual monetary value of these extra costs. Applying the multiplier to the poverty line only yields an approximate monthly monetary value based on goods and services that are not disability specific. To gain a deeper understanding of the factors driving these extra costs for disability, we turn to the results from the GS and GSR studies.

### 4.2 GSR FGD result

The initial GSR FGDs with disability experts provide a comprehensive list of disability-specific goods and services specifically tailored to meet the needs of people with disabilities (Table 5). For the purposes of this study, these disability-specific goods and services are priced at premium rate due to their specialized nature and are not currently covered by existing government support programs.

**Table 5:** Scope of goods and services used and required by persons with disability for participation, summary of Indonesia GSR FGDs, 2021.

Type	Findings
Assistive devices	◦ Cost to possess these devices consist of product price, fitting cost, running and maintenance costs (batteries, spare parts), and additional accommodation and transport should the buyer live far from the assistive device provider. ◦ A wide range of products are used depending on type and severity of disability. Many rely on multiple assistive products and/or use both conventional and non-conventional, such as smartphone apps for text and voice recognition. ◦ Many assistive devices (including hearing aids, braille displays, video magnifiers, magnifier software, screen readers, scanners) must be imported and therefore are subject to additional tax and customs related charges. ◦ National Health Insurance (JKN) covers only 7 conventional assistive devices and quality is variable.
Human assistance	◦ People with several forms of disability require continuous human assistance, like people with hearing difficulties would need sign language support, people with self-care difficulties need assistant/caretaker. ◦ Very costly (direct provision) and/or opportunity cost (if family member must leave work to provide care).
Transportation	◦ Many people with disability cannot use public transport because infrastructure is not disability-friendly. ◦ The commonly used forms are costly, namely modified private vehicles and semi-private vehicles (taxis or online taxis).
Health	◦ Major expense as many persons with disability are without JKN or private insurance. ◦ JKN covers several types of health treatments (physiotherapy & decubitus medication) but not other recurring preventative treatments. ◦ Rehabilitation treatment for persons with psychosocial and self-care disability is not covered by JKN.
School	◦ Only special schools provide inclusive educational environment, learning process, curriculum etc. ◦ Some non-specialist schools run an inclusive education system. But parents must usually pay out of their own pocket for special support such as a shadow teacher, sign language interpreter or a specialist teacher.
Work	Some offices, such as government agencies, can fund sign language interpreters and make the workplace more accessible. But this is still rare.

Summary of FGDs with experts representing 8 types of disability – seeing, mobility, upper body function, multiple disability, self-care, remembering/concentrating, hearing and communicating. October-November 2020.

### 4.3 GS survey and GSR validation result

Results from the GS survey were then used to construct a monthly monetary cost for people with disabilities. The survey managed to gather a total of 388 different cost drivers that can be classified into 6 main categories of cost: assistive devices; special accommodation; human assistance/help; intrastate/commute travel; interstate travel; and essential needs. The monthly cost of each category was derived by generating a monthly equivalent of cost to access items under each category^3^. Since most of these drivers are all disability-specific cost, we may regard the obtained cost as extra cost of disability.

The monthly costs are presented in disability type-disaggregated cost and overall cost of having disability (averaged through all types of disability). These costs are presented in three main ways—the median, mean and 75th percentile (referred to as P75). The median represents the lower end of the disability cost, the mean represents the middle value and the P75 the higher end^4^. To ensure the validity of the results, they were also discussed in the validation FGDs with experts. Overall, the experts agree with the obtained values, including the overall cost of disability. However, there was an exception regarding the cost of assistive devices, where the experts believed the values should be much higher.

### 4.4 Result triangulation from the three methods

The GS survey results support the previous finding from SOL approach, indicating that different types of functioning difficulties incur varying levels of extra costs. Mobility and self-care difficulties tend to incur higher costs compared to other types of difficulties (Supplementary Figure S7). However, the survey also revealed an important nuance: households with multiple types of disabilities experience higher costs compared to households with a single type of disability. This finding challenges our previous approach in the SOL analyses, where we applied a single type of multiplier to households with multiple types of disabilities. Therefore, we need to reconsider our treatment for multiple types of disabilities in order to more accurately capture the associated costs.

In addition, we were also interested in assessing if people with disabilities could afford these costs. We approached the question through two main methods. Firstly, we compared the disability type-specific costs with Yogyakarta province's minimum wage. By considering the minimum wage as a benchmark for maintaining a basic standard of living, these analyses provided insights into the affordability of disability costs for individuals with disabilities who earn the minimum wage. Additionally, we aggregated all the disability median costs and compared against wealth distribution of residents in Yogyakarta province to provide a clearer understanding of whether individuals across the consumption distribution the financial means had to afford these costs. We limited our analyses to Yogyakarta province, as the site of the data collection.

The findings indicate that in Yogyakarta province, the monthly costs of disability generally exceed the minimum wage when considering the mean and P75 values, and only surpass the median for certain types of disabilities (Supplementary Figure S8). The largest gap between expenditure and the minimum wage is observed among individuals with multiple disabilities whereas those with remembering/concentrating disabilities face the smallest gap.

Moreover, it is important to note that even the minimum general cost of disability is too expensive for most people. This finding is based on a comparison between the median general monthly cost of disability from the GS survey, regardless of disability type, with the cumulative per capita expenditure distribution in Yogyakarta province from Susenas data, March round of year 2019. The cost is estimated at around IDR 1.86 million (≈USD 133 in November 2020 rate), or equal to the expenditure level of persons from the 80th percentile of IDR 1.87 million (Supplementary Figure S8). To put it in simpler terms, only households within the top 20% of the wealth distribution are financially capable of covering the costs associated with disability.

The required goods and services for individuals with disabilities also vary by demographic profile. Young people with disability (below 25 years old) had to face monthly costs between IDR 1.7 million to IDR 5.35 million (≈USD 121–382) for education purposes. The key cost drivers among high payers in this group are shadow/supplementary teachers, specialized health professionals and assistive devices for educational purposes.

Productive-age people with disability (ages 25–59), face costs of IDR 4 million–24.8 million (≈USD 286–1,771) for work purposes. Travel expenses, especially commuting, play a significant role as individuals in this age group who are employed tend to spend more on transportation. Other cost drivers for productive-age individuals include assistive devices for mobility and self-care difficulties, human assistance, and accessible infrastructure.

For elderly individuals with disabilities (ages 60+), the main cost drivers are assistive devices related to self-care; travel expenses (intra-state/commute); and the need for human assistance^5^. The latter is particularly important as elderly people with disabilities often require assistance to carry out their daily activities.

The study highlights that the extra costs associated with disability are substantial and vary by disability type and age group, affecting various aspects of life such as work, education, and daily self-care. The findings indicate that most individuals, including those earning a minimum wage, cannot afford these costs, emphasizing the need for support and interventions that alleviate the financial burden faced by people with disabilities.

From a policy perspective, SDG Target 1.3 and its corresponding indicator 1.3.1 emphasize the importance of establishing and expanding thesocial protection system to provide support for the poor and vulnerable populations, including individuals with disabilities. The findings of this study have policy implications that align with the SDG framework. Specifically, the findings emphasize the importance of considering the adjusted poverty line for individuals with disabilities when designing poverty targeted interventions. This is crucial as many individuals who are actually living in poverty may not be identified without these adjustments. Furthermore, considering the widespread issue of unaffordable costs, it is crucial to implement complementary interventions that extend support beyond a narrow scope, ensuring a more inclusive approach to social protection. Recognizing the variation in expenses across different age groups, it is essential to develop a tailored “package of programs” instead of adopting a one-size-fits-all approach. The subsequent section will delve into how the extra costs study results have been leveraged to inform the design of “disability concession” package.

## 5 Leveraging extra costs study findings to inform Indonesia’s policy framework on disability concessions

The study findings on extra costs have played a pivotal role in shaping a comprehensive policy framework for “disability concessions” in Indonesia. Measuring the extra costs illuminates the financial barriers faced by Indonesian with disabilities, and the provision of concessions is designed to alleviate these costs. It is worth noting that the concept of concessions is relatively new in Indonesia, with its fiscal implications yet to be fully understood. The study findings, in tandem with situational analysis, international benchmarking and insights derived from consultations with OPDs, collectively establish foundational evidence that informs the relevant policymakers. These findings contribute a set of well-defined recommendations, offering clear entry points and modality for assessing the fiscal impact of the novel approach. This section of the paper aims to elucidate how the study results have been instrumental in defining the scope and structure of these concessions, starting with the alignment with policy goals, the design of concession package, and sector-specific insights from the study.

The provision of concessions has been mandated under the Law No. 8/2016 on Persons with Disabilities. The 2016 Law signifies a significant shift towards promoting disability rights and inclusivity in Indonesia, departing from the Law No. 4/1997 which based on medical- and charity-based approaches. The 2016 Law defines disability more broadly and emphasizes empowerment, with mandates extending to various sectors. In this context, “disability concessions” refer to specific benefits provided to individuals with disabilities to alleviate the extra costs and barriers associated with disabilities.

Concession is a novel policy approach under Indonesia social security scheme. The current provisions for people with disability was given in forms of health and employment insurance scheme, alongside a poverty-tested social assistance scheme. The proposed concessions aim to complement these existing programs and be offered to all persons with disabilities, acknowledging that not all individuals may be eligible for cash allowances but still require assistance to partially compensate for the additional costs of living with disabilities (25, 26).

The findings of the extra cost analyses played a crucial role in justifying each benefit proposed in the concession package (refer to Supplementary Figure S4). The study findings on extra costs were utilized to prioritize the provision of concessions, taking into account benchmarking with concession provided by other countries and consultations with organizations of persons with disabilities. The proposed package of coordinated concession spans four main sectors: education, health, transportation, and utilities. Examples of benefits include disability specific top-ups on education allowances, premium subsidies and additional coverage for assistive devices and disability rehabilitation in health, ticket discounts for interstate and intrastate/commute travels in transportation, and discounts on tap water, electricity, and internet in utilities. Each benefit is delivered to meet the 20% discount from the actual costs, with the discount amount is based on estimates from the standard of living approach that the poorest individuals with more severe disabilities spend up to 20% more per capita on essential goods.

### 5.1 Concession recommendations for health sector

In the health sector, the main rationale was the high current costs and out-of-pocket expenses faced by individuals with disabilities. The study revealed that respondents had to spend approximately IDR 80,000–735,000 (equal to USD 5.71–52.5 in 2020 currency rate) per visit to access appropriate health services. It was also found that persons with psychosocial and self-care disabilities require regular preventative and rehabilitative treatment, but many are unable to access these services due to their high costs or unavailability.

Assistive devices emerged as one of the significant cost drivers in health expenses, with majority not covered by Indonesia's national health insurance program (or JKN). These devices play a critical role in enabling daily activities and basic participation for individuals with disabilities. Moreover, the expense associated with assistive devices is not limited to a one-time purchase, as they often require ongoing maintenance and additional running costs, such as batteries and spare parts. Importantly, many of these devices, including hearing aids, braille displays, video magnifier software, and screen readers, are exclusively imported, subjecting them to additional import-related taxes and fees.

Insights from the GS survey and GSR FGDs shed light on the extensive range of costs associated with assistive devices, spanning from IDR 20,000 (=USD 1.4) for basic sanitary products addressing self-care difficulties to IDR 150 million (=USD 10,714.3) for a cochlear implant specifically designed for individuals with severe disabilities. However, what emerged as particularly striking was the discovery that 60% of the participants in the goods and services survey, conducted among people with disabilities, reported not using any assistive devices at all. The primary reasons cited were the high costs of these devices, their unavailability in the market, or a lack of awareness of their existence.

These findings underscored the significant value of integrating individual interviews and focus group data on the present usage of assistive devices with a market survey that focuses on prioritized assistive products recommended by the World Health organization. This market survey was carried out after the extra cost survey while our team was concurrently involved in designing concessions. By combining these diverse sources of information, we were able to generate a more accurate estimate of the costs of assistive products and estimate the costs of including them through the national health insurance.

### 5.2 Concession recommendations for education sector

The study recommends prioritizing students with disabilities in accessing the Program Indonesia Pintar (PIP), a renowned scholarship program aimed at supporting economically disadvantaged students and providing them with supplementary support. It is concerning that in the past three years, 70% of individuals with moderate to severe disabilities either lack any formal education or have merely completed primary schooling. The GS survey and GSR FGDs shed light on the financial burden that students with disabilities who strive to continue their education face. On average, these students spend approximately 1.7 million per month on supplementary teachers and education-related expenses due to the school's inability to meet their individualized needs. In other countries like Nepal, offering supplementary support such as residential services, travel assistance and specialized learning materials, alongside investments in inclusive education, has proven to significantly enhance enrolment and academic performance for students with disabilities.

### 5.3 Concession recommendations for utilities sector

There are two justifications for offering concession on utilities (electricity, internet and tap water). Firstly, both the SOL and GSR FGDs suggest that households with individuals with disabilities bear higher expenses related to utilities compared to those without disabilities. For instance, households with mobility and self-care disabilities are more likely to purchase clean water compared to households without disabilities. Individuals with hearing, vision and communication difficulties often rely on specialize mobile phone applications for their daily activities, resulting in additional internet costs due to their disability status.

Secondly, offering concessions on electricity, internet packages and water transport can provide an effective means to reach a broader population and ensure equal benefits. Unlike other proposed concession sub-programs that inevitably limit the number of beneficiaries, such as transport discounts targeting individuals with disabilities in urban areas where public transport more readily available, discounts on utilities have the potential to benefit a wider range of individuals. Since most people already incur these costs, offering discounts on utilities has the advantage of reaching a larger proportion of the population.

### 5.4 Concession recommendations for transport sector

The study recommends the provision of discounted tickets for both interstate and intrastate commute/travels. This measure aims to enhance mobility and increase the economic participation of persons with disabilities by reducing the financial burden associated with transportation expenses.

## 6 Discussion

The SDG framework serves as a valuable tool in monitoring progress towards disability inclusion, encompassing key areas such as poverty, education, and decent work. While the SDG framework acknowledges the need for policy solutions that expand social protection to include people with disabilities, there is a need for better insights and tailored policy approaches to address the underlying challenges. This paper emphasizes integrating the concept of “extra costs of living” with disabilities within the SDG framework. The study, conducted in collaboration between the Indonesian Ministry of Finance and the Australia-Indonesia Partnership for Economic Development, employs three methods to measure these costs and inform the design of appropriate “concessions”.

The findings from Standard of living approach reveal that households with moderate to severe disabilities incur higher per capita extra cost, reaching up to 20% for each type of disability. The poorest households (or lowest consumption quintile groups) cannot afford the additional costs for basic needs compared to wealthier groups. When adjusting the poverty line to account for extra costs, the presence of disability significantly increases the risk of poverty, with nearly 1 in 4 households with higher threshold disability (i.e., moderate to severe difficulties) being considered poor, compared to 1 in 12 for households with no disabilities. This is in line with other country-level studies like Cambodia (27), Bosnia Herzegovina (28), Mongolia (29) and Ghana (30), with all demonstrated that incorporating the disability costs to the standard poverty line significantly increases the proportion of poor people with disabilities.

The goods and services and goods and services required method complements the SOL approach, showing that different types of disabilities entail varying levels of extra costs, with mobility and self-care disabilities having higher costs. Households with multiple types of disabilities experience higher costs than those with single type. The monthly costs of disability in Yogyakarta province generally exceed the minimum wage, making them unaffordable for individuals with disabilities earning at that level. Furthermore, the findings also indicate that only the richest 20% of the population can afford the estimated disability-related monthly expenses, underscoring the significant financial burden faced by individuals and families with disabilities. Finally, the goods and services required for individuals with disabilities vary depending on their age group. Young people with disabilities require significant costs for education-related expenses, productive age face costs related to work, and elderly individuals for self-care and daily activities.

We faced challenges during survey implementation. We purposively recruited enumerators with disabilities or experience working with people with disabilities to ensure effective communication and understanding. As the data collection coincided with the pandemic, their prior experience enabled us to conduct face-to-face surveys while strictly following social distancing measures. However, these enumerators had limited experience with conducting socio-economic surveys and struggled with roster-type questions and complex skipping patterns. Establishing clear communication with them was also difficult as all training and cross-checking had to be done online, increasing the time and effort required for the study. Many of the disability-specific goods and services included in the questionnaire were unfamiliar to the respondents, resulting in enumerators spending additional time explaining them. As result, the interviews took longer than anticipated, leading to respondent fatigue and a significant amount of missing information.

However, the enumerator team deserves credit for revisiting respondents to clarify and complete missing information as far as possible. While these issues were partly due to the unprecedented circumstances of the pandemic, the main lessons learned is the need to revisit and streamline data collection instruments and investment effort in enumerator training and common understanding between the research team and enumerators.

Despite these efforts, the reported expenses for accessing assistive devices were significantly underestimated, as many respondents and experts were unaware of the devices required, such as screen readers and braille displays for individuals with vision difficulties. The few devices that were reported to be used were relatively inexpensive, such as diapers for self-care or basic sticks and canes for enhanced mobility. As a result, the annual median cost estimates for assistive devices commonly used was very low, around Rp50,000 (≈USD 3.57 using November 2020 exchange rate). The validation FGDs expressed disappointment with these results but couldn't provide a credible alternative. Therefore, we supplemented the study with a separate market survey of assistive devices recommended by the WHO (31) to be provided through the national health insurance system. The findings from this supplementary study will be presented in a forthcoming paper. In this paper, we will only present the results of the GSR study.

The study's findings offer valuable insights into areas where concessions are needed, including in education, health, transportation, and utilities, to alleviate burden of accessing essential goods and services for individuals and families. Future studies can explore diverse approaches to utilize the information on extra costs of disability to inform policy and program actions. Implementing the study presented challenges, including the lack of comprehensive database on people with disabilities, hindering random respondent selection and limiting the sample size. The survey implementation was also affected by the COVID-19 pandemic, requiring strict adherence to social distancing measures and online enumerator training. Enumerators encountered difficulties with complex survey questions and unfamiliarity with disability specific goods and services. These issues resulted in longer interviews, respondent fatigue and missing information. The reported expense for assistive devices were significantly underestimated, necessitating further research on assistive devices to inform the concession design. Key lessons learned for future studies include the importance of revising and streamlining data collection instruments and investing in enumerator training to ensure common understanding between the research team and enumerators.

To conclude, the study provides new insights that can inform policy action to address the financial barriers and improve the wellbeing of individuals and families with disabilities, thereby contributing to the realization of SDG's vision of “leaving no one behind”.

## Data Availability

The original contributions presented in the study are included in the article/Supplementary Material, further inquiries can be directed to the corresponding author.

## Footnotes

^1^The latest figure being 60.14% in 2023, with the 2019 or pre-pandemic share being 56.64% (19).

^2^The basic needs consist of 52 representing food items to meet daily calorie requirements of 2,100 kCal, and 51 non-food items in urban areas or 47 in rural to meet housing, apparel, education and health needs (24).

^3^To generate this monthly cost equivalent, we first extract the costs and basis of frequency (e.g., per trip, per week, perpetual and lasts for around how long, etc) from the survey. Then we extracted the frequency of use for disability-specific goods and services from the initial GSR FGD, and for general goods and services from the use of non-disabled people (therefore serving the purpose of “required frequency”). The cost to access assistive devices is derived from the perpetual cost or expenses made equivalent to one-year subscription. Human assistance/help was already served as monthly cost. Interstate/commute travel's cost is collected per trip, yet each respondent is deemed to do around 20 round travels for work/school per month and socialise at least 4 times per month. Intrastate travel is deemed to be around 2 round trips annually.

^4^Mean is the direct average of all respondents who report the cost. Median is the middle value reported from the survey, that is relatively free from the extreme ends of the data and therefore more neutral. The P75 is the third quartile value reported from the survey, showing how much is generally spent by the respondents who can afford more diverse goods and services for disability support.

^5^We do not report the range of cost for this age group due to low survey representation from the elderly age groups and lower than expected cost range.

## Appendix

**Technical explanation for Standard of Living (SOL) Approach**

For our econometric exercise, the household income is replaced with the natural logarithm value of per capita expenditure, deflated with Indonesian provincial poverty line. The expenditure is shown as per capita to better measure individual consumption. We also convert the expenditure into real terms by standardizing them with official provincial urban-rural poverty line released by Statistics Indonesia. This is done to remove the spatial and temporal variation from the expenditure. Poverty line is used in lieu of consumer price index to deflate the expenditure, because Indonesian poverty line is designed to capture the same basket of goods across Indonesia consumed by the reference population, therefore serving a purpose for standardization. The limitation of using this alternative measure is its apparent bias towards the bottom of the economic distribution and does not reflect well the needs of higher income households (15). However, this also suggests that the extra cost result we obtain will better capture the needs of people who may need support the most. Lastly, the expenditure is presented in logarithmic form to transform the skewed distribution of expenditure to be normally distributed.

We define the standard of living by constructing a normalized tetrachoric asset index made out of household ownership of 16 asset items and private dwelling structures (Table A1). To obtain this value, first each household is assigned 1 if they own the corresponding asset/infrastructure and 0 if not. Afterwards, we check the interrelationship of all variables used with Cronbach's Alpha score to determine internal consistency, check for their shared pattern using tetrachoric correlation, and shortlist the most relevant variables based on the results. We aim to get a strong correlation score, preferably above 0.7, and include a list of variables regularly available in the other Susenas waves. Afterwards, we proceeded to create an asset index by conducting a principal component analysis out of the selected variables using the tetrachoric correlation matrix we obtained during the variable testing and predict the index by using just the first component. Lastly, the obtained index value range is then normalized to 0–100 before utilizing it in SOL econometric exercises.

**Table A1:** Comparison of asset index specification, 2019.

Specification	Cronbach's alpha score	Correlation with natural log of real per capita expenditure	Remarks
Number of assets owned (4 variables), normal correlation	0.0394	0.2325	
Asset & private dwelling structure (16 binary variables), normal correlation	0.7572	0.5941	Highest correlation
Asset & private dwelling structure (16), tetrachoric correlation		**0** **.** **5893**	**Chosen measure**
Asset, private dwelling structure, & Food Insecurity Experience Scale/FIES (24), normal correlation	0.8021	0.4457	
Asset, private dwelling structure, & FIES (24), tetrachoric correlation		0.5304	

Author's calculations using Susenas 2019, March wave.

During the asset index calculation, we also conducted several robustness tests. We considered additional measures included in the questionnaire, such as number of assets owned, and also considered a combination of asset, infrastructure, and Food Insecurity Experience Scale (FIES). To assess the shared ordinality, we test the correlation value between the normalized indices derived from said combination of variables with an expenditure measure. Out of the three calculated indices, the one using assets and private dwelling structure ownership based on normal correlation had the highest correlation at 0.5941 compared to the others (Table 3). However, the use of normal correlation is not advised as the strictly binary variables used to construct the asset index might be indicative of an underlying latent variable that could potentially skew the result (32). Furthermore, the difference in correlation between the normal-correlation index and tetrachoric index is not that significant. Thus, we made the decision to use assets and private dwelling structure ownership with tetrachoric correlation instead.

In the calculation process, some of the variables are automatically reversed by the calculation engine to ensure consistency. Out of the 26 assets and 3 basic infrastructure ownership asked in the questionnaire, we ended up using 13 assets and 3 private dwelling structure which shared the most internal consistency and has a similar shared correlation pattern (Table A2).

**Table A2:** Variables that defined the final asset index, 2019.

Asset ownership variable	Weight
Liquid petroleum gas tank of at least 5.5 kg capacity	28.3%
Refrigerator	28.9%
Air conditioner	32.1%
Water heater	24.6%
Landline phone	27.9%
Laptop	30.2%
Gold/golden jewellery (at least 10 g)	26.2%
Car	30.9%
Flat TV	29.1%
Land	16.3%
Cell phone	23.7%
Savings	25.8%
Second house	16.8%
Access to improved water	14.5%
Access to improved sanitation	18.4%
House with good dwelling materials	14.9%
Common variance explained	0.4686

Weight is based on the first component of principal component analysis using tetrachoric correlation.

Author's calculations using Susenas 2019, March wave.

For the vector of other characteristics, we chose several variables that explain the household demographics, which are area of residence (urban/rural), characteristics of household head (sex, age, age squared, and years of schooling), dependency ratio within household (young and old), and working member ratio within household. The regression was done on household level, and after several attempts we found that running the regression separately by type of disability and expenditure quintile produced the most stable result. The complete result of the econometric exercise is available in Table A3.

**Table A3:** Summary of regressions for calculating extra cost, 2019.

(A) Slight, moderate, and severe disability											
Variables	Aggregated	Sight					Hearing				
		Q1	Q2	Q3	Q4	Q5	Q1	Q2	Q3	Q4	Q5
Ln real per capita expenditure	14.7605***	11.1987***	10.9841***	12.6584***	16.1719***	15.3774***	11.1795***	10.9750***	12.6230***	16.1002***	15.3906***
Disability status	−0.5609***	−0.3294*	−0.4749**	−0.7599***	−1.1968***	−1.9144***	−0.5240**	−0.6205**	−1.1365***	−1.5203***	−2.2036***
Urban/rural	4.5532***	3.6902***	4.4927***	5.2404***	5.0302***	6.0801***	3.6888***	4.4967***	5.2461***	5.0476***	6.1061***
Province dummy (33 dummies)	Included	Not included	Not included	Not included	Not included	Not included	Not included	Not included	Not included	Not included	Not included
Male HH head	−0.4211***	0.0381	0.3321	0.213	−0.3086	−0.0982	0.0405	0.3322	0.2313	−0.2838	−0.056
HH size	2.8297***	1.7386***	2.2486***	2.7731***	3.5800***	4.8497***	1.7388***	2.2516***	2.7749***	3.5809***	4.8499***
Age of HH head	0.6829***	0.3370***	0.4695***	0.5505***	0.6618***	0.8562***	0.3312***	0.4614***	0.5383***	0.6454***	0.8330***
Square age of HH head	−0.0044***	−0.0021***	−0.0032***	−0.0039***	−0.0045***	−0.0053***	−0.0020***	−0.0031***	−0.0038***	−0.0043***	−0.0051***
Years of schooling of HH head	1.2855***	0.7887***	0.9122***	1.1350***	1.3889***	1.6847***	0.7883***	0.9117***	1.1343***	1.3902***	1.6892***
Young dependency ratio of HH head	3.5768***	−2.7478***	−0.2028	1.2129**	2.7621***	3.5724***	−2.7689***	−0.1959	1.2389**	2.8144***	3.6770***
Old dependency ratio of HH head	−2.4216***	−5.3385***	−5.1273***	−3.8060***	−4.3065***	−4.2139***	−5.2953***	−5.1070***	−3.7478***	−4.3152***	−4.2990***
Working HH member ratio	−3.4255***	−1.0103***	−1.0381***	−2.1633***	−2.4369***	−3.6908***	−1.0379***	−1.0435***	−2.1505***	−2.4366***	−3.6482***
Constant	−215.6211***	−147.0933***	−152.3792***	−181.1987***	−238.6156***	−240.2229***	−146.6984***	−152.0889***	−180.4810***	−237.3325***	−240.1504***
R-squared	0.5868	0.2974	0.3152	0.3699	0.4331	0.5591	0.2975	0.3152	0.3699	0.4329	0.5586
Observations	1204466	327303	259585	240162	206905	155699	327303	259585	240162	206905	155699
**Extra cost**	**3.8%**	**2.9%**	**4.3%**	**6.0%**	**7.4%**	**12.4%**	**4.7%**	**5.7%**	**9.0%**	**9.4%**	**14.3%**

Variables	Mobility					Remembering/concentrating				
	Q1	Q2	Q3	Q4	Q5	Q1	Q2	Q3	Q4	Q5
Ln real per capita expenditure	11.1961***	10.9656***	12.6491***	16.1556***	15.4053***	11.1865***	10.9705***	12.6508***	16.1409***	15.3868***
Disability status	−0.1873	−0.3421	−0.0829	−1.3716***	−1.8242***	−0.3355	−1.2673***	−1.4814***	−1.5551***	−2.7654***
Urban/rural	3.6923***	4.5006***	5.2621***	5.0600***	6.1135***	3.6926***	4.4913***	5.2431***	5.0513***	6.1079***
Province dummy (33 dummies)	Not included	Not included	Not included	Not included	Not included	Not included	Not included	Not included	Not included	Not included
Male HH head	0.045	0.3375	0.2373	−0.3172	−0.112	0.0382	0.2983	0.2041	−0.308	−0.1023
HH size	1.7331***	2.2452***	2.7619***	3.5810***	4.8530***	1.7349***	2.2643***	2.7810***	3.5815***	4.8580***
Age of HH head	0.3355***	0.4665***	0.5494***	0.6511***	0.8386***	0.3347***	0.4583***	0.5372***	0.6516***	0.8343***
Square age of HH head	−0.0021***	−0.0032***	−0.0039***	−0.0044***	−0.0051***	−0.0021***	−0.0031***	−0.0038***	−0.0044***	−0.0051***
Years of schooling of HH Head	0.7893***	0.9129***	1.1373***	1.3905***	1.6889***	0.7889***	0.9108***	1.1343***	1.3898***	1.6876***
Young dependency ratio of HH head	−2.7214***	−0.1764	1.3337**	2.7532***	3.5932***	−2.7449***	−0.3458	1.1577*	2.7764***	3.6253***
Old dependency ratio of HH head	−5.3828***	−5.1531***	−3.9165***	−4.2850***	−4.2278***	−5.3633***	−5.0127***	−3.7171***	−4.3182***	−4.2729***
Working HH member ratio	−1.0097***	−1.0414***	−2.1020***	−2.5231***	−3.7256***	−1.0261***	−1.1391***	−2.2305***	−2.4804***	−3.7234***
Constant	−147.0355***	−152.0821***	−181.1305***	−238.1617***	−240.3914***	−146.8691***	−151.8508***	−180.7509***	−237.9637***	−240.0241***
R-squared	0.2973	0.315	0.3695	0.4329	0.5587	0.2973	0.3158	0.3703	0.433	0.559
Observations	327303	259585	240162	206905	155699	327303	259585	240162	206905	155699
**Extra cost**				**8.5%**	**11.8%**		**11.6%**	**11.7%**	**9.6%**	**18.0%**

Variables	Communicating					Self-care				
	Q1	Q2	Q3	Q4	Q5	Q1	Q2	Q3	Q4	Q5
Ln real per capita expenditure	11.1878***	10.9702***	12.6235***	16.1319***	15.3988***	11.2083***	10.9529***	12.6374***	16.1635***	15.4081***
Disability status	−0.238	−1.4517***	−1.1697***	−2.1655***	−3.4049***	0.0955	−1.2534***	−0.5352	−1.2347**	−3.0471***
Urban/rural	3.6941***	4.5005***	5.2585***	5.0616***	6.1244***	3.6945***	4.4969***	5.2616***	5.0593***	6.1106***
Province dummy (33 dummies)	Not included	Not included	Not included	Not included	Not included	Not included	Not included	Not included	Not included	Not included
Male HH head	0.0485	0.3263	0.2311	−0.2909	−0.0526	0.0555	0.3452	0.236	−0.275	−0.0661
HH size	1.7312***	2.2557***	2.7674***	3.5769***	4.8511***	1.7285***	2.2517***	2.7646***	3.5733***	4.8515***
Age of HH head	0.3357***	0.4613***	0.5443***	0.6523***	0.8397***	0.3369***	0.4630***	0.5483***	0.6583***	0.8387***
Square age of HH head	−0.0021***	−0.0031***	−0.0038***	−0.0044***	−0.0052***	−0.0021***	−0.0032***	−0.0039***	−0.0045***	−0.0052***
Years of schooling of HH Head	0.7892***	0.9114***	1.1359***	1.3914***	1.6904***	0.7896***	0.9138***	1.1372***	1.3931***	1.6922***
Young dependency ratio of HH head	−2.7091***	−0.3041	1.2501**	2.7926***	3.6354***	−2.6486***	−0.2555	1.3044**	2.8613***	3.6449***
Old dependency ratio of HH head	−5.4153***	−5.1214***	−3.8695***	−4.4219***	−4.3800***	−5.4500***	−5.1531***	−3.9030***	−4.4331***	−4.3990***
Working HH member ratio	−0.9984***	−1.1109***	−2.1694***	−2.4817***	−3.6927***	−0.9499***	−1.1219***	−2.1378***	−2.4411***	−3.7259***
Constant	−146.9352***	−151.9531***	−180.6008***	−237.8894***	−240.3879***	−147.2779***	−151.7949***	−180.9240***	−238.5118***	−240.4958***
R-squared	0.2973	0.3156	0.3698	0.433	0.5588	0.2973	0.3154	0.3696	0.4326	0.5587
Observations	327303	259585	240162	206905	155699	327303	259585	240162	206905	155699
**Extra cost**		**13.2%**	**9.3%**	**13.4%**	**22.1%**		**11.4%**		**7.6%**	**19.8%**

Variables	Multi disability				
	Q1	Q2	Q3	Q4	Q5
Ln real per capita expenditure	11.1741***	10.9750***	12.6268***	16.1224***	15.3844***
Disability status	−0.5020**	−1.2114***	−1.0002***	−1.6773***	−2.8647***
Urban/rural	3.6908***	4.4815***	5.2412***	5.0462***	6.0843***
Province dummy (33 dummies)	Not included	Not included	Not included	Not included	Not included
Male HH head	0.0234	0.2867	0.2045	−0.3311	−0.1361
HH size	1.7412***	2.2659***	2.7759***	3.5894***	4.8665***
Age of HH head	0.3333***	0.4579***	0.5407***	0.6449***	0.8277***
Square age of HH head	−0.0021***	−0.0031***	−0.0038***	−0.0043***	−0.0050***
Years of schooling of HH head	0.7881***	0.9107***	1.1343***	1.3883***	1.6835***
Young dependency ratio of HH head	−2.8209***	−0.4248	1.1617*	2.6744***	3.5339***
Old dependency ratio of HH head	−5.2647***	−4.9017***	−3.7101***	−4.2259***	−4.1256***
Working HH member ratio	−1.0772***	−1.1955***	−2.2328***	−2.5481***	−3.7915***
Constant	−146.6283***	−151.8512***	−180.4995***	−237.5166***	−239.7671***
R-squared	0.2975	0.316	0.37	0.4333	0.5594
Observations	327303	259585	240162	206905	155699
**Extra cost**	**4.5%**	**11.0%**	**7.9%**	**10.4%**	**18.6%**

(B) Moderate and severe disability.											
Variables	Aggregated	Sight					Hearing				
		Q1	Q2	Q3	Q4	Q5	Q1	Q2	Q3	Q4	Q5
Ln real per capita expenditure	14.7605***	11.1922***	10.9750***	12.6510***	16.1785***	15.3925***	11.1787***	10.9706***	12.6375***	16.1672***	15.4036***
Disability status	−0.5609***	−0.5457*	−0.9992**	−1.5164***	−2.6290***	−3.4500***	−0.9314***	−1.0307**	−1.3923**	−1.5031**	−3.3810***
Urban/rural	4.5532***	3.6937***	4.4943***	5.2551***	5.0485***	6.1238***	3.6915***	4.4976***	5.2606***	5.0577***	6.1291***
Province dummy (33 dummies)	Included	Not included	Not included	Not included	Not included	Not included	Not included	Not included	Not included	Not included	Not included
Male HH head	−0.4211***	0.0448	0.3434	0.2305	−0.2848	−0.0648	0.0489	0.3441	0.2385	−0.2729	−0.0571
HH size	2.8297***	1.7334***	2.2443***	2.7664***	3.5722***	4.8441***	1.7353***	2.2459***	2.7648***	3.5686***	4.8375***
Age of HH head	0.6829***	0.3352***	0.4666***	0.5467***	0.6540***	0.8494***	0.3319***	0.4644***	0.5453***	0.6560***	0.8463***
Square age of HH head	−0.0044***	−0.0021***	−0.0032***	−0.0039***	−0.0044***	−0.0053***	−0.0020***	−0.0032***	−0.0039***	−0.0045***	−0.0053***
Years of schooling of HH head	1.2855***	0.7888***	0.9126***	1.1357***	1.3904***	1.6905***	0.7885***	0.9129***	1.1361***	1.3926***	1.6923***
Young dependency ratio of HH head	3.5768***	−2.7258***	−0.1709	1.2603**	2.8643***	3.6774***	−2.7456***	−0.1597	1.3012**	2.9050***	3.7075***
Old dependency ratio of HH head	−2.4216***	−5.3880***	−5.1805***	−3.8640***	−4.4159***	−4.3796***	−5.3603***	−5.1914***	−3.8877***	−4.4688***	−4.4261***
Working HH member ratio	−3.4255***	−1.0101***	−1.0431***	−2.1716***	−2.4368***	−3.6219***	−1.0247***	−1.0177***	−2.1317***	−2.3858***	−3.6264***
Constant	−215.6211***	−146.9713***	−152.2058***	−181.0338***	−238.6001***	−240.4981***	−146.7187***	−152.1236***	−180.8567***	−238.5453***	−240.6258***
R-squared	0.5868	0.2974	0.3152	0.3698	0.433	0.5586	0.2975	0.3151	0.3697	0.4325	0.5584
Observations	1204466	327303	259585	240162	206905	155699	327303	259585	240162	206905	155699
**Extra cost**	**3.8%**	**4.9%**	**9.1%**	**12.0%**	**16.2%**	**22.4%**	**8.3%**	**9.4%**	**11.0%**	**9.3%**	**21.9%**

Variables	Mobility					Remembering/concentrating				
	Q1	Q2	Q3	Q4	Q5	Q1	Q2	Q3	Q4	Q5
Ln real per capita expenditure	11.2023***	10.9644***	12.6556***	16.1741***	15.4052***	11.2001***	10.9629***	12.6131***	16.1380***	15.4005***
Disability status	−0.0664	−0.6676*	0.3981	−1.4191***	−3.0253***	−0.1227	−1.0641**	−2.2204***	−2.1848***	−2.8689***
Urban/rural	3.6943***	4.4993***	5.2636***	5.0585***	6.1187***	3.6943***	4.5009***	5.2628***	5.0585***	6.1246***
Province dummy (33 dummies)	Not included	Not included	Not included	Not included	Not included	Not included	Not included	Not included	Not included	Not included
Male HH head	0.0533	0.3477	0.2433	−0.287	−0.0721	0.0513	0.3363	0.2248	−0.2836	−0.0671
HH size	1.7300***	2.2437***	2.7576***	3.5744***	4.8439***	1.7304***	2.2466***	2.7735***	3.5754***	4.8420***
Age of HH head	0.3365***	0.4669***	0.5513***	0.6570***	0.8408***	0.3365***	0.4663***	0.5441***	0.6554***	0.8476***
Square age of HH head	−0.0021***	−0.0032***	−0.0039***	−0.0045***	−0.0052***	−0.0021***	−0.0032***	−0.0038***	−0.0045***	−0.0053***
Years of schooling of HH head	0.7896***	0.9136***	1.1374***	1.3928***	1.6910***	0.7895***	0.9133***	1.1356***	1.3922***	1.6921***
Young dependency ratio of HH head	−2.6770***	−0.1697	1.3867**	2.8361***	3.6348***	−2.6835***	−0.1908	1.1928**	2.8264***	3.6964***
Old dependency ratio of HH head	−5.4332***	−5.1925***	−3.9625***	−4.4266***	−4.3889***	−5.4322***	−5.1988***	−3.8702***	−4.4504***	−4.4313***
Working HH member ratio	−0.9756***	−1.0464***	−2.0573***	−2.4476***	−3.7149***	−0.9798***	−1.0518***	−2.2021***	−2.4422***	−3.6417***
Constant	−147.1703***	−152.0841***	−181.2935***	−238.6203***	−240.4711***	−147.1347***	−152.0357***	−180.4287***	−238.0713***	−240.5891***
R-squared	0.2973	0.3151	0.3695	0.4326	0.5587	0.2973	0.3152	0.3701	0.4327	0.5584
Observations	327303	259585	240162	206905	155699	327303	259585	240162	206905	155699
**Extra cost**		**6.1%**		**8.8%**	**19.6%**		**9.7%**	**17.6%**	**13.5%**	**18.6%**

Variables	Communicating					Self-care				
	Q1	Q2	Q3	Q4	Q5	Q1	Q2	Q3	Q4	Q5
Ln real per capita expenditure	11.1992***	10.9831***	12.6340***	16.1445***	15.4033***	11.2157***	10.9724***	12.6540***	16.1669***	15.4017***
Disability status	−0.1322	−2.1337***	−1.4827**	−2.6441***	−2.5969*	0.4135	−0.5487	0.3286	−0.8209	−2.8400***
Urban/rural	3.6943***	4.5062***	5.2627***	5.0643***	6.1294***	3.6952***	4.5008***	5.2625***	5.0585***	6.1218***
Province dummy (33 dummies)	Not included	Not included	Not included	Not included	Not included	Not included	Not included	Not included	Not included	Not included
Male HH head	0.0523	0.3389	0.2383	−0.281	−0.0577	0.0568	0.3507	0.2413	−0.2736	−0.0575
HH size	1.7300***	2.2501***	2.7638***	3.5742***	4.8397***	1.7274***	2.2420***	2.7591***	3.5691***	4.8412***
Age of HH head	0.3366***	0.4642***	0.5478***	0.6598***	0.8527***	0.3366***	0.4677***	0.5503***	0.6618***	0.8488***
Square age of HH head	−0.0021***	−0.0032***	−0.0039***	−0.0045***	−0.0054***	−0.0021***	−0.0032***	−0.0039***	−0.0045***	−0.0053***
Years of schooling of HH Head	0.7895***	0.9116***	1.1367***	1.3925***	1.6933***	0.7896***	0.9136***	1.1374***	1.3939***	1.6934***
Young dependency ratio of HH head	−2.6793***	−0.237	1.3043**	2.8554***	3.6944***	−2.6272***	−0.1344	1.3646**	2.9037***	3.6720***
Old dependency ratio of HH head	−5.4378***	−5.2442***	−3.9385***	−4.4692***	−4.4351***	−5.4624***	−5.2318***	−3.9437***	−4.4707***	−4.4316***
Working HH member ratio	−0.9745***	−1.0699***	−2.1362***	−2.4218***	−3.6272***	−0.9328***	−1.0103***	−2.0768***	−2.3790***	−3.6590***
Constant	−147.1298***	−152.2395***	−180.8666***	−238.2814***	−240.7521***	−147.3800***	−152.2358***	−181.2355***	−238.6753***	−240.6363***
R-squared	0.2973	0.3156	0.3697	0.4328	0.5583	0.2973	0.315	0.3695	0.4324	0.5584
Observations	327303	259585	240162	206905	155699	327303	259585	240162	206905	155699
**Extra cost**		**19.4%**	**11.7%**	**16.4%**	**16.9%**					**18.4%**

Variables	Multi disability				
	Q1	Q2	Q3	Q4	Q5
Ln real per capita expenditure	11.1916***	10.9676***	12.6354***	16.1718***	15.3965***
Disability status	−0.2926	−1.4039***	−0.8144*	−2.0664***	−3.4741***
Urban/rural	3.6941***	4.4928***	5.2602***	5.0581***	6.1134***
Province dummy (33 dummies)	Not included	Not included	Not included	Not included	Not included
Male HH head	0.0483	0.3382	0.2349	−0.2853	−0.0605
HH size	1.7321***	2.2504***	2.7662***	3.5776***	4.8469***
Age of HH head	0.3355***	0.4625***	0.5473***	0.6521***	0.8401***
Square age of HH head	−0.0021***	−0.0031***	−0.0039***	−0.0044***	−0.0052***
Years of schooling of HH head	0.7892***	0.9122***	1.1368***	1.3918***	1.6907***
Young dependency ratio of HH head	−2.7177***	−0.265	1.2735**	2.8082***	3.6486***
Old dependency ratio of HH head	−5.4132***	−5.1685***	−3.8951***	−4.4353***	−4.4272***
Working HH member ratio	−1.0076***	−1.1168***	−2.1568***	−2.4826***	−3.6953***
Constant	−146.9791***	−151.9597***	−180.8581***	−238.4624***	−240.3505***
R-squared	0.2973	0.3154	0.3696	0.4328	0.5587
Observations	327303	259585	240162	206905	155699
**Extra cost**		**12.8%**	**6.4%**	**12.8%**	**22.6%**
