# Supplementary material for: Counting the costs: understanding the extra costs of living with disability in Indonesia to advance inclusive policies within the SDG framework
Source: Front Rehabil Sci. 2024 Oct 22;5:1236365. doi: 10.3389/fresc.2024.1236365 (PMC11534708; doi:10.3389/fresc.2024.1236365)
Supplement: Supplementary file 1 [file Datasheet1.docx]

Supplementary Material

Counting the costs: Understanding the extra costs of living with disability in Indonesia to advance inclusive policies within the SDG framework

Irma Marlina, Ginanjar Wibowo, Desi Dwi Bastias, Dinar Dwi Prasetyo, Bimbika Sijapati Basnett, and Mercoledi Nasiir*

*** Correspondence:** Corresponding Author: [Mercoledi.Nasiir@prospera.or.id](mailto:Mercoledi.Nasiir@prospera.or.id)

# Supplementary Figures


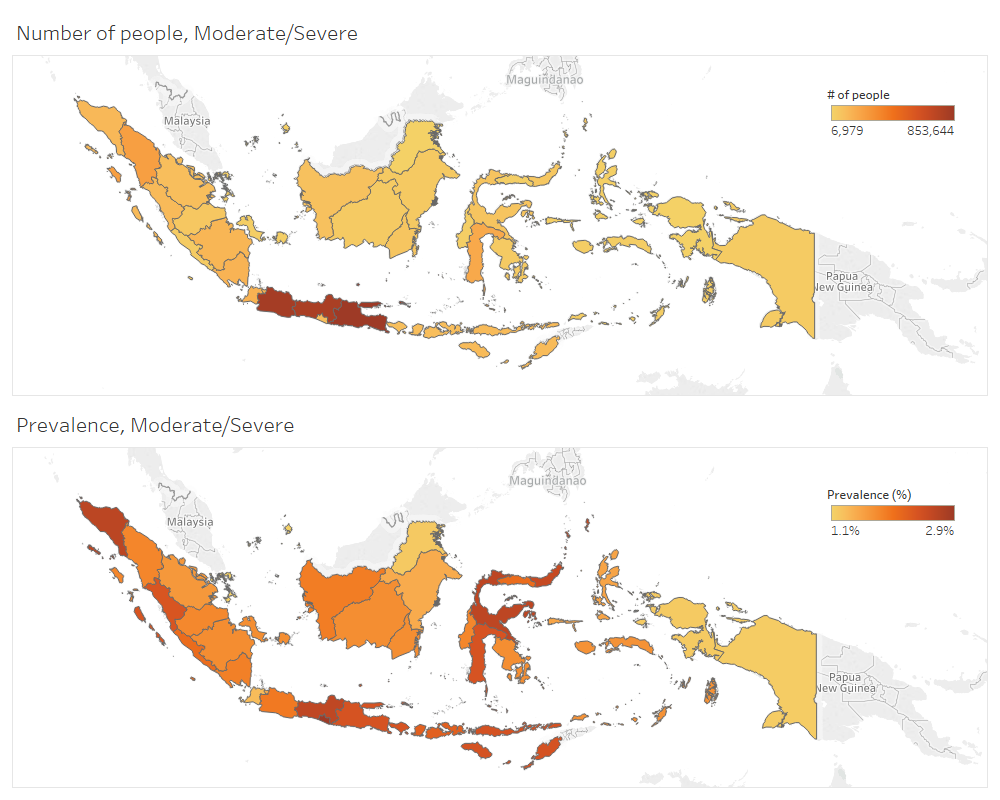


**Supplementary Figure 1.** Prevalence of Moderate/Severe Disability in Indonesia by province, 2015. Source: Author’s calculation using Supas 2015

**Supplementary Figure 2.** Estimated formal education survival rate by disability status, ages 25-35, 2022. Source: Author’s calculation from Susenas data, March round, 2022

Supplementary Figure 3. Labour force flow chart by disability status, 2022. Source: Author’s calculation using Sakernas data, August round, 2022

**Supplementary Figure 4.** Estimated poverty rate by disability status, 2018-2022. Source: Author’s calculation using Susenas data, March round, 2018-2022

Supplementary Figure 5. Poverty Rate by Disability Status, using Official and Adjusted Poverty Line, 2019. Source: Author’s calculation, using Susenas data, March round, 2019

Supplementary Figure 6. Monthly Cost by Type as opposed to Yogyakarta Minimum Wage, 2020. Source: Author’s calculation using results from GS survey, SIGAB-Prospera, 2021

Supplementary Figure 7. Median Disability-related Cost, in comparison with Yogyakarta Expenditure Percentile (Susenas 2019). Source: Author’s calculation using results from GS survey, SIGAB-Prospera, 2021 and Susenas data, March round, 2019, BPS.


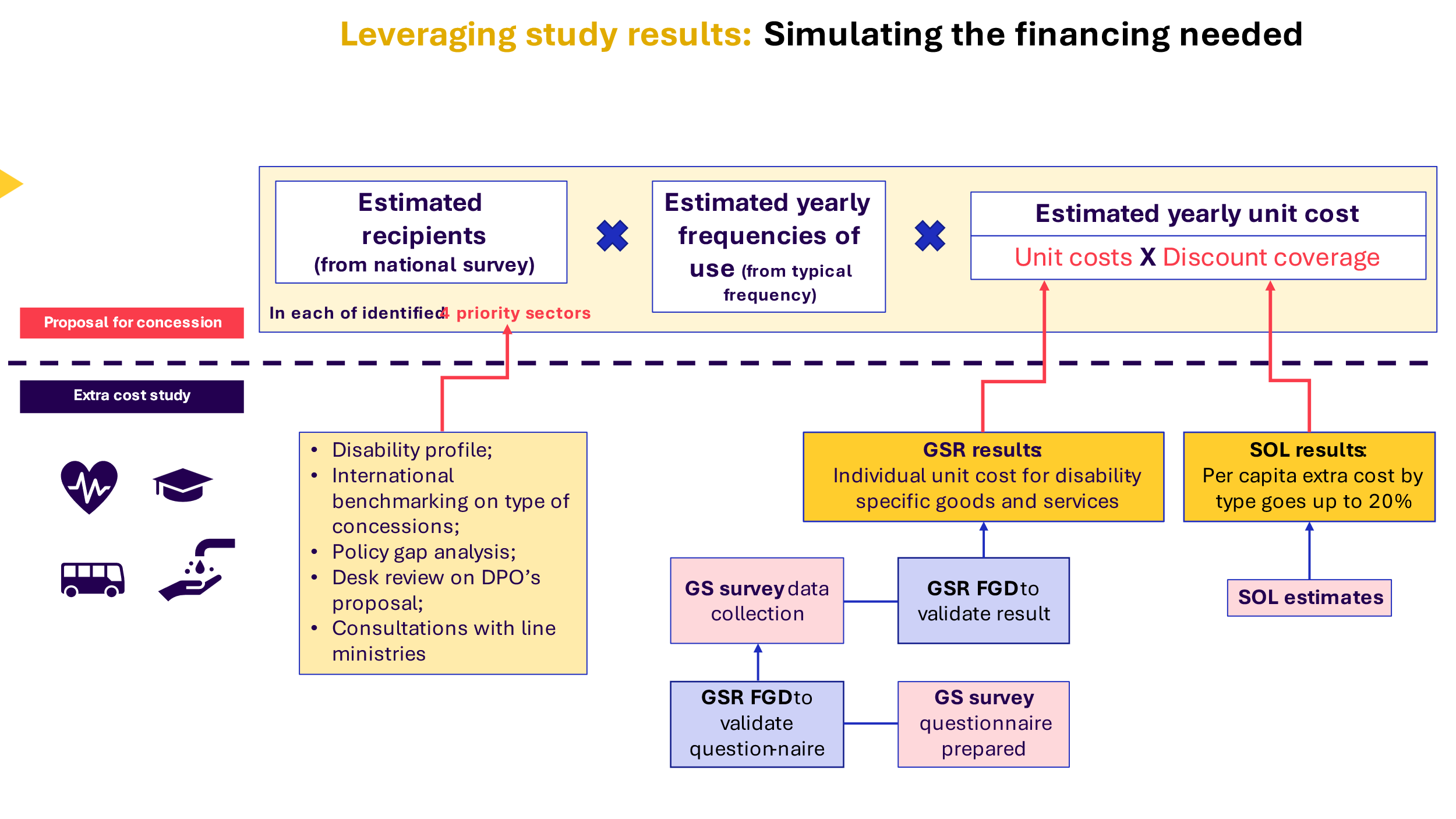


**Supplementary Figure 8.** Leveraging extra cost study results for informing national disability concession program.
